# Should acupuncture become a complementary therapy in the treatment of uterine fibroid: a systematic review and meta-analysis of randomized controlled trials

**DOI:** 10.3389/fmed.2023.1268220

**Published:** 2023-12-13

**Authors:** Yuehan Ren, Junning Zhang, Weizhen Wu, Yi Yuan, Jiale Wang, Yi Tang, Yan Liao, Xinmin Liu

**Affiliations:** ^1^Department of Gynaecology, China Academy of Chinese Medicine Sciences Guang’anmen Hospital, Beijing, China; ^2^Graduate School, Beijing University of Chinese Medicine, Beijing, China; ^3^Department of Oncology of Integrative Chinese and Western Medicine, China-Japan Friendship Hospital, Beijing, China

**Keywords:** acupuncture, uterine fibroid, complementary therapy, systematic review, meta-analysis

## Abstract

**Background:**

Uterine fibroids (UFs) are the most common benign tumors in women of reproductive age. The most effective treatment is myomectomy, but there is no long-term or low-invasive treatment option exists. Acupuncture can be used to treat UFs in a variety of ways. However, there is no meta-analytic synthesis including valid data that explored the efficacy of acupuncture for UFs.

**Objective:**

To assess the efficacy and safety of acupuncture for treating UFs.

**Methods:**

The PRISMA 2020 checklist was used. We identified and extracted the trials through may 2023 from six databases. The quality of the trials was assessed using the risk of bias (2.0). Meta-analysis was performed using RevMan 5.4 software, and it was synthesized using the random-effects model if the included studies were in high heterogeneity. Subgroup and sensitivity analysis were used if necessary.

**Results:**

A total of 1,035 trials were identified, of which 11 were included in the review and meta-analysis. In terms of acupuncture scheme design and fibroid-related symptoms, the trials are highly heterogeneous. All 11 trials have reported acupuncture types, with traditional acupuncture and electroacupuncture being the more representative subgroups. A qualitative review of existing evidence shows that acupuncture has no serious adverse reaction on UFs. Meta-analysis shows that acupuncture can effectively reduce the volume of UFs (MD – 3.89, 95% CI – 5.23 to – 2.56, *P* < 0.00001) or uterine volume (MD – 16.22, 95% CI – 19.89 To – 12.55, *p* < 0.00001), reduce the score of fibroid symptoms (MD – 3.03, 95% CI − 3.45 to – 2.60, *p* < 0.00001), improve the treatment efficiency (RR: 0.19, 95% CI: 0.13 to 0.25, *p* < 0.00001), and likely do not affect the estrogen level.

## 1 Introduction

Uterine fibroids (UFs), also known as leiomyomas, are benign tumors that arise from the smooth muscle tissue layer (myometrium) of the uterus (1). They are the most common benign tumors in women of reproductive age. The prevalence of UFs is estimated to be between 4.5–80% depending on study populations and diagnostic methods (2–5). Accompanying a high incidence rate, the substantial direct and indirect costs to healthcare payers and society are large. Some studies (6, 7) show that the total costs range from $11,717–25,023 per patient per year, after diagnosis or surgery among patients with UFs.

The exact cause of UFs is unknown, but several risk factors have been identified, including age, race, obesity, and family history. It is worth mentioning that those aged 41–50 or 51–60 years were 10 times more likely to have UFs than those aged 21–30 years (2). Symptoms of UFs can include heavy menstrual bleeding, pelvic pain or pressure, iron deficiency anemia, or urinary frequency, which make a huge impact on women’s quality of life (8).

Treatment options for UFs, such as uterine artery embolization, myomectomy, surgical removal of the uterus (hysterectomy) and high-frequency magnetic resonance-guided focused ultrasound surgery (MRgFUS) (9). However, there are currently no low-invasive treatments that are simple, inexpensive, and effective enough and fertility-preservation (1, 10, 11). Traditional Chinese Medicine (TCM) may provide an alternative option for patients with UFs.

Acupuncture is a key modality based on TCM theory and has been used for thousands of years to treat a wide range of conditions (12, 13). One of the unique features of acupuncture is that it is minimally invasive and has very few side effects when performed by a qualified practitioner. It is also a holistic therapy that aims to treat the underlying causes of disease, rather than just the symptoms. Acupuncture can be used to treat a wide range of conditions, which are commonly used to support fertility and reproductive health (14, 15), as well as to promote overall wellness and balance in the body.

One possible mechanism of acupuncture-treated UFs is through the modulation of the hypothalamic–pituitary-ovarian (HPO) axis (16, 17). The HPO axis plays a critical role in regulating female reproductive function, and it has been linked to the development of UFs (18). Acupuncture has been shown to regulate the HPO axis by modulating the release of gonadotropin-releasing hormone (GnRH) and other hormones involved in reproductive function (19). Another possible mechanism is through the modulation of local blood flow. UFs are known to be highly vascularized (20), and acupuncture has been shown to increase blood flow to the uterus and surrounding tissues (21). This increased blood flow may help to reduce the size and symptoms of UFs. Finally, acupuncture may also have an anti-inflammatory effect. Inflammation has been implicated in the development and progression of UFs, and acupuncture has been shown to reduce levels of pro-inflammatory cytokines in the body (22–25).

Due to the lack of summary evidence for the acupuncture treatment of UFs, we conducted a systematic review and meta-analysis to evaluate the effectiveness and safety of acupuncture treatment of UFs, and to provide evidence-based medicine evidence for clinical decision-makers and guide-makers.

## 2 Methods

### 2.1 Standards followed and registration information

The PRISMA (Preferred Reporting Items for Systematic Reviews and Meta-Analyses) 2020 checklist was followed. This review had been registered on PROSPERO (ID: CRD42023428921). Considering the actual research situation, some information has been modified.

### 2.2 Criteria for considering studies

#### 2.2.1 Types of studies

Randomized controlled trials (RCTs), whether blinded or not, were included.

#### 2.2.2 Types of participants

Patients diagnosed as UFs according to any authoritative clinical guidelines, such as Chinese expert consensus on the diagnosis and treatment of UFs (26).

#### 2.2.3 Types of interventions

In our review, the interventions of the treatment group include acupuncture such as traditional acupuncture, electroacupuncture, wrist-ankle acupuncture, abdominal acupuncture, warm acupuncture and moxibustion (a method of fixing moxa sticks on acupuncture needles which seen as a holistic form of therapy) and fire needle therapy.

Comparison interventions include sham acupuncture, traditional Chinese medicine, western medication and no intervention. Both groups of patients could receive routine care. The difference in intervention mode between the two groups was only the specific acupuncture. Trials evaluating acupuncture that made use of nonpenetrating point stimulation, such as acupressure and transcutaneous electrical nerve stimulation (TENS), and trials comparing various acupuncture techniques, were disregarded by the research team.

#### 2.2.4 Types of outcome measures

Primary outcomes were UFs volume or uterine volume. Secondary outcomes included: (i) Quality of life measured using validated scales including The UFs Symptom and Health-Related Quality of Life (UFS-QOL) or other validated TCM syndrome scales; (ii) Estradiol levels during menstruation; (iii) The total effective rate. Improvement of clinical signs and symptoms, and 30% or more reduction of UFs are considered effective; (iv) Safety of the acupuncture intervention including adverse events and withdrawals for any reason.

### 2.3 Literature search strategy

Six databases were searched from inception to May 23rd, 2023 to identify potentially relevant articles with no restrictions on language. The databases included China National Knowledge Infrastructure (CNKI), Wanfang Database for Chinese Technical Periodicals, PubMed, Web of Science, Embase, and Cochrane Central Registry of Controlled Trials (CENTRAL). The key search terms were (fibroid* or leiomyoma* or fibromyoma* or fibroma*) and (acupuncture or electroacupuncture or acupoint or needling or needle or warm acupuncture and moxibustion or fire needle or wrist-ankle acupuncture). This search contained both MESH and Non-MESH terms. All searches were limited to studies on humans. Reference lists of full text papers were searched, and any relevant articles identified were screened.

### 2.4 Study selection and data extraction

The screening was carried out separately by YHR and JNZ. After screening the titles and abstracts of every trial that was searched, the complete texts of any publications that could be pertinent were retrieved and read. Discussions between the two reviewers were used to settle disagreements, occasionally with the help of a third reviewer (WZ W).

Data from included trials were extracted and double-entered into a database for analysis. The following information was extracted and recorded on a standardized data extraction form: publication information (first author, year, title, country), participant characteristics, study design (sample size, age, UFs diagnosis criteria, group assignment, control condition), acupuncture protocol (acupoint formula, technique, treatment session, duration, and frequency), outcomes, time points for assessments, and adverse events. When inadequate data were supplied in the full text, authors were contacted through e-mail to provide extra information on trial details.

### 2.5 Risk of bias assessment

Two reviewers (YHR and JNZ) independently assessed the risk of bias using the methods endorsed by The Cochrane Collaboration. This tool appraises trials in seven domains: Random sequence generation (selection bias), Allocation concealment (selection bias), Blinding of participants and personnel (performance bias), Blinding of outcome assessment (detection bias), Incomplete outcome data (attrition bias), Selective reporting (reporting bias), Other bias (acupuncture scheme design bias). Each domain is rated as low risk (of bias), high risk, or unclear risk. Any issues were settled by discussion. The specific detail please review Cochrane Handbook for Systematic Reviews of Interventions.^1^

### 2.6 Acupuncture treatment regimen

Each trial’s acupuncture treatment regimen was analyzed using the Standards for Reporting Interventions in Clinical Trials of Acupuncture (STRICTA) (27). The STRICTA checklist includes acupuncture rationale, details of needling, treatment regimen, other components of treatment, practitioner background, and comparator interventions.

### 2.7 Statistical analysis

The meta-analysis was performed using Cochrane Collaboration Review Manager (RevMan 5.4). The continuous data were presented with the mean difference (MD) with 95% confidence intervals (CIs). The dichotomous data were presented using relative risk (RR) with 95% CIs. We analyzed the efficacy differences between therapies by pooling the results of all trials. The I2 statistic was used to assess statistical heterogeneity between trials (28). I2 values of 50% or less indicate no observable heterogeneity, while I2 values of more than 50% indicate significant heterogeneity (29). The random-effects model would be used to synthesize a meta-analysis if there is no significant heterogeneity among the included trials. If heterogeneity was high, we used subgroup analysis or sensitivity analysis to manage data.

## 3 Results

### 3.1 Identification of studies

**Figure 1:**
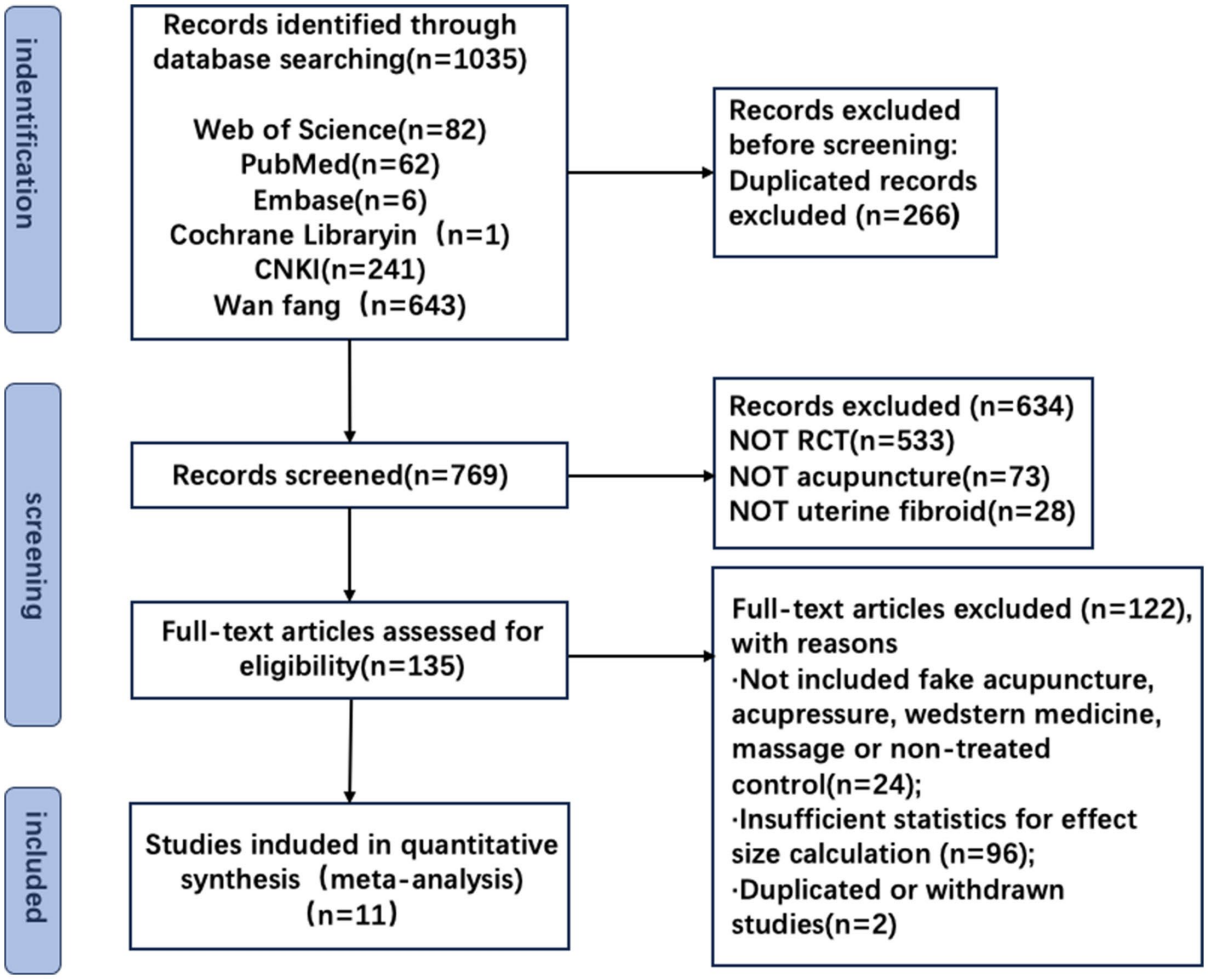
Flowchart of the literature review and selection process.

The search yielded 1,035 potential titles and abstracts for review, of which 266 were duplicates and 634 were excluded due to ineligible conditions. A total of 135 full-text articles were reviewed for further assessment, of which 122 were excluded for various reasons (see Figure 1). The remaining 11 trials (30–40) were included in our review.

The summarized characteristics are listed in Table 1. Eleven trials were all published in the Chinese language. Sample size ranged from 60 (31, 32, 37, 39) to 84 (36). The included trials comprised 735 UFs patients (mean age 34 to 48 years). There were 5 participants reported to have withdrawn from included trial (33).

**Table 1:** Information of the included studies.

No.	Study ID	Sample size (Acup/ Control)	age(Acup/ Control)	Diagnosis for inclusion	Intervention (regimen)	Control (regimen)	Course of treatment	Number of treatments	Outcomes	Adverse events
1	Guo 2016 (30)	80(40/40)	42/42	UE	WAM (N.r.)	Juli Sanjie Pill	3 months	N.r.	MV, ER	N.r.
2	Huang 2019 (31)	60(30/30)	42.19 ± 3.82/42.73 ± 3.94	UE	EA (3/week)	Shaofu Zhuyu Decoction	3 months	36	MV, ER	N.r.
3	Liu 2011 (32)	60(30/30)	40.06 ± 6.51/40.16 ± 6.94	UE & Qi stagnation and Blood stasis pattern	TA (1/day for the first week, 1/week for the last)	Fukangle capsule	3 months	18	MV, EL, ER	N.r.
4	Liu 2016 (33)	61(30/31)	34.97 ± 7.67/35.90 ± 7.91	UE & Qi stagnation and Blood stasis pattern	TA (1/day)	Xiangleng Pill	6 weeks	42	MV; SS; ER	N.r.
5	Peng 2010 (34)	62(32/30)	38.6 ± 7/39 ± 6.8	UE	TA (N.r.)	Self formulated decoction	2 months	N.r.	MV; ER	N.r.
6	Wan 2013 (35)	64(32/32)	38.5	UE	EA (1/day for 15 days and 7 days rest, periodicity)	CM from Pattern differentiation	3 months	66	MV	N.r.
7	Xie 2021 (36)	84(42/42)	38 ± 6/38 ± 6	UE	TA (1/day)	Mifepristone	1 months	30	MV, UV, ER, IAR	7 in TA: headache (2); gastrointestinal reactions(3); hot flash(1); abdominal pain(1) 5 in Mifepristone: headache(1); gastrointestinal reactions(3); hot flash(1).
8	Yang 2019 (37)	60(30/30)	47.8 ± 5.1/48.2 ± 4.5	UE	TA (1/day)	Huoxue Xiaozheng Decoction	42 days	42	MV; SS; ER; IAR	No adverse events occurred
9	Zhang 2020 (38)	64(32/32)	42.48 ± 4.02/41.42 ± 3.96	UE	TA (1/day)	Huoxue Xiaozheng Decoction	42 days	42	MV, UV, EL, SS	N.r.
10	Zhang 2013 (39)	60(30/30)	42.77 ± 5.12/42.33 ± 4.27	UE & Blood stasis pattern	CET (1/month)	Guizhi Fuling capsule	3 months	3	MV, UV, SS, ER	Mild nausea(6); aseptic inflammation(3)
11	Zhou 2018 (40)	80(40/40)	37.69 ± 2.14/35.88 ± 2.26	UE	TA (3/week)	Guizhi Fuling capsule	3 months	40	MV, ER	N.r.

WAM, warm acupuncture and moxibustion; EA, Electroacupuncture; TA, traditional acupuncture; CET, Catgut-embedding therapy; UE, Ultrasonic examination; CM, Chiness Medicine; N.r., not reported; MV, myoma volume; UV, Uterine volume; EL, Estrogen level; SS, Symptom score; ER, Effective rate; IAR, Incidence of adverse reactions.

### 3.2 Diagnosis of UFs

All 11 trials enrolled women with UFs and used imaging criteria to determine the diagnosis. Four of the trials (36–38, 40) highlighted clinical symptoms and gynecological tests as supplementary diagnostic tools, such as stomach pain, bladder compression, increased menstrual volume, and longer cycles. Two trials (32, 33) met the criteria for syndrome differentiation of qi stagnation and blood stasis while meeting the diagnosis of UFs, and one trial (39) meet the criteria for syndrome differentiation of blood stasis, as formulated by the Ministry of Health of China in the Guiding Principles for Clinical Research of New Drugs of Traditional Chinese Medicine for UFs.

### 3.3 Acupuncture interventions

Of the 11 trials, seven (32–34, 36–38, 40) involved 176 participants using traditional acupuncture, two (31, 35) involved 62 participants using electroacupuncture, one (30) involved 42 participants using warm acupuncture, one (39) involved 30 patients using catgut-embedding therapy (Table 1).

### 3.4 Control interventions

Among the 11 interventions studied, 9 trials used fixed TCM formulas, including Juli Sanjie Pill (30), Shaofu Zhuyu Decoction (31), Fukangle Capsule (32), Xiangling Pill (33), Huoxue Xiaozheng Decoction (37, 38), Guizhi Fuling Capsule (39, 40), and Self formulated decoction (34). One trial (35) selected prescriptions based on syndrome differentiation. One trial (36) used mifepristone (Table 1).

Four trials (30, 32, 33, 39) have set up two control groups. The interventions of the experimental group for these four trials are acupuncture combined with TCM. Only if the control group with only this TCM can show the efficacy of acupuncture. Our review only retained this control group and ignored another control group, which included blank control (39), simple acupuncture (33), mifepristone (30), and unrelated TCM (32).

### 3.5 Risk of bias in included trials

**Figure 2:**
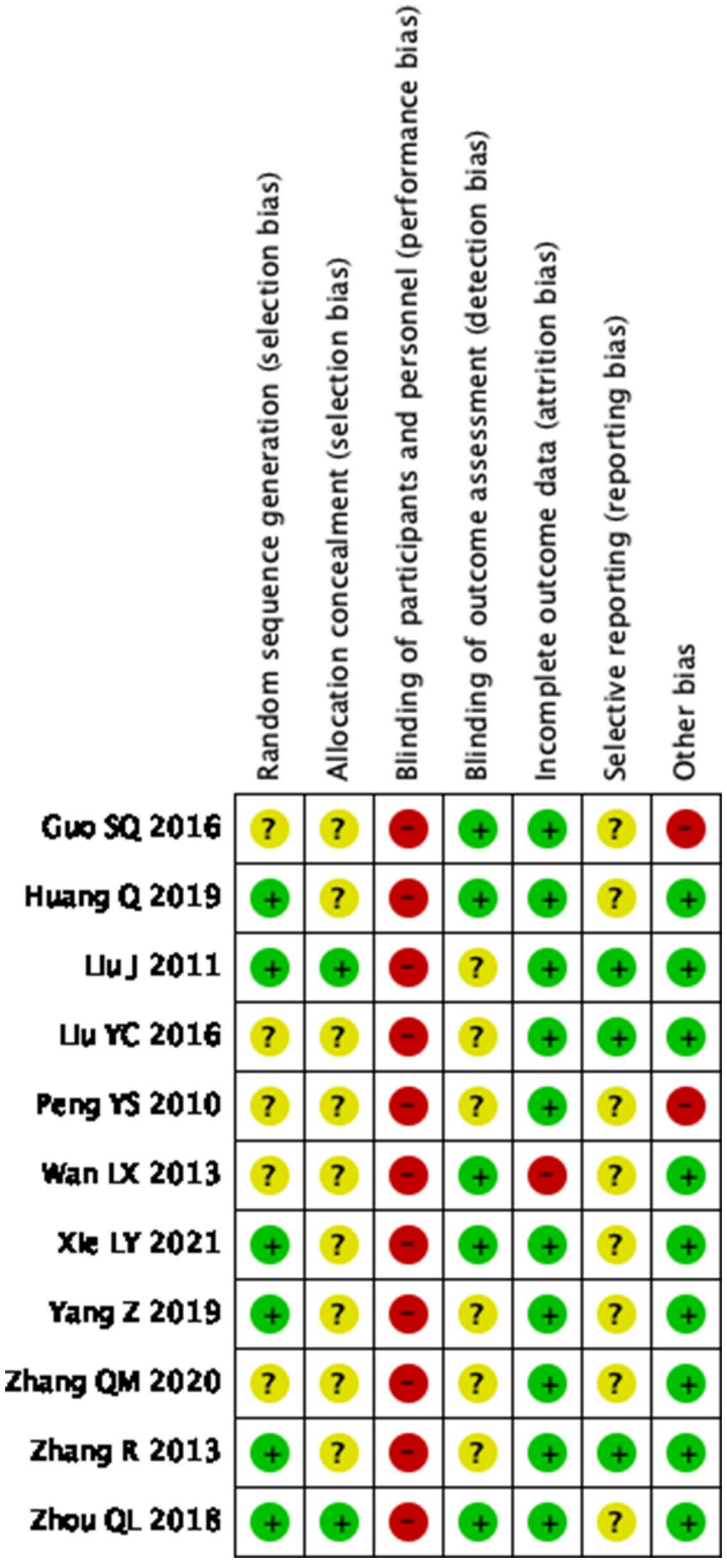
Risk of bias domain for each included study.

**Figure 3:**
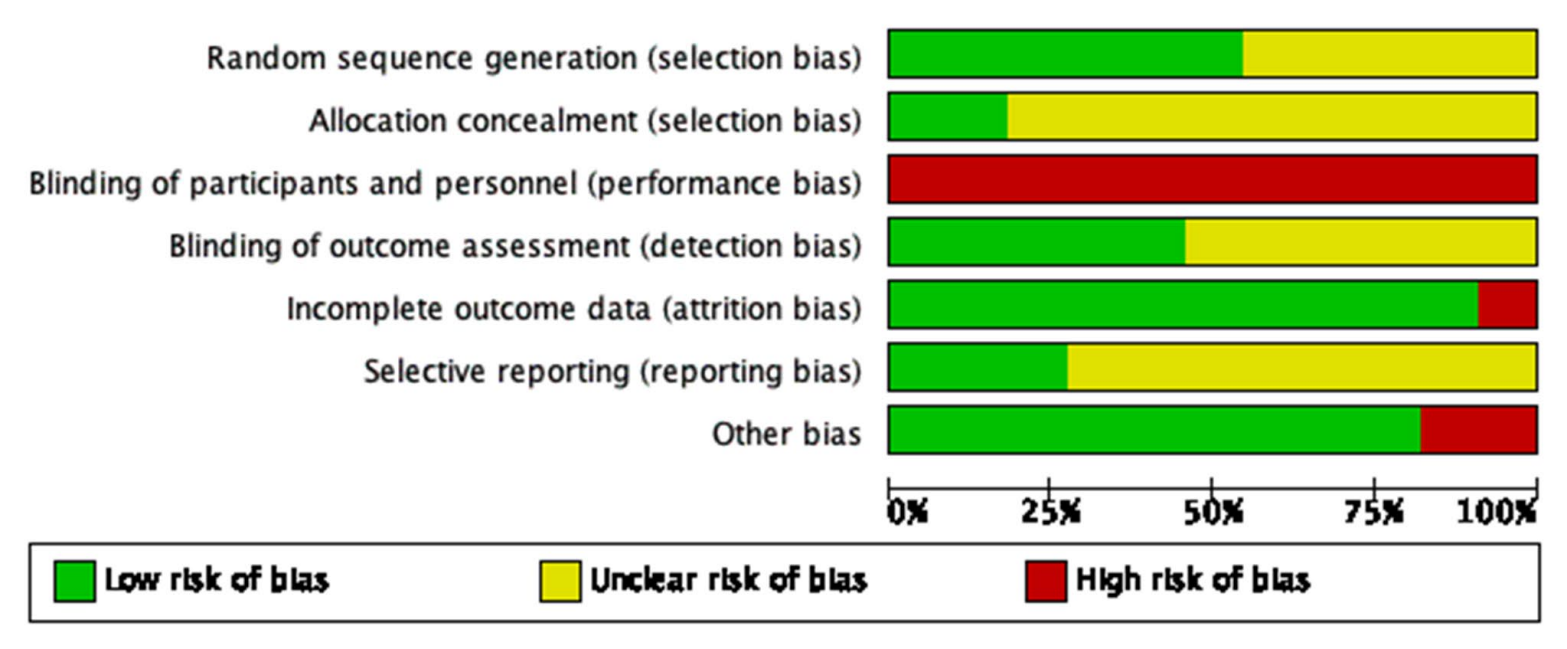
Risk of bias summary.

Figure 2 shows the various biases included in the trials. A graphical summary of the bias risk assessment conducted by the authors based on 7 bias risk areas for the review of 11 included trials is shown in Figure 3. Score 2 points for each item that is high risk, 1 point for items that are ambiguous, and 0 points for those that are low risk. A total score of 1–8 represents little danger, while a score of 9–12 represents high risk. In all domains, no experiment has been judged as having a minimal risk of bias. Our investigation included 11 trials that were all categorized as low risk.

#### 3.5.1 Randomization

Six trials (studies 2,3,7,8,10,11) were at low risk of bias for randomization. They mentioned implementation-specific randomization methods, such as random number tables, or random grouping with sequentially numbered, sealed, opaque envelopes. Five trials (studies 1,4,5,6,9) did not describe the method of randomization which was assessed as unclear.

#### 3.5.2 Allocation

Two trials (studies 3,11) were assessed as low risk of bias for allocation concealment. Jing Liu et al. used a random number table as the method of assigning concealment. Qinglian Zhou et al. use random envelopes to assign concealment. The remaining 9 trials (studies 1,2,4,5,6,7,8,9,10) did not report the method of allocation, so we assessed their risk as unclear.

#### 3.5.3 Blinding

11 trials involved acupuncture versus oral medication so that participants could not be blinded. We assessed a total of 11 trials as having a high risk for performance bias. Most trials used clinician-rated outcome measures. Five trials (studies 1,2,6,7,11) were rated as having a low risk of detection bias and the remaining six (studies 3,4,5,8,9,10) did not report on blinding of the assessor/clinician, the analyst, or outcome measures, and were rated as unclear risk.

#### 3.5.4 Incomplete outcome data

The outcome indicators for trials with only fibroid volume or uterine volume (study 6) are assessed as high-risk. Trials that included other secondary outcome indicators were (studies 1,2,3,4,5,7,8,9,10,11) rated as low risk.

#### 3.5.5 Selective reporting

Eight trials (studies 1,2,5,6,7,8,9,11) were rated as unclear risk, owing to no study protocol or trial registration records being available. Three trials (studies 3,4,10) did report data on all included outcomes and were at low risk of bias.

#### 3.5.6 Other potential sources of bias

In 9 trials (studies 2,3,4,6,7,8,9,10,11), we rated the risk from other biased sources as low. In two trials (studies 1,5), we evaluated the irrationality of the design of experiments such as acupuncture treatment course and frequency, and these trials were rated as high risk.

### 3.6 Outcome measurement

Results of 11 trials were summarized to evaluate the clinical efficacy of acupuncture in the treatment of UFs. The volume of UFs was used as the main therapeutic index in 11 trials, the volume of uterus was reported in 3 trials (36, 38, 39), and the estrogen level was included in 2 trials (32, 38). Four trials used a quality-of-life assessment scale, including the UFS-QOL scale (37, 38) and the validated TCM syndrome score (33, 39). Nine trials (30–34, 36, 37, 39, 40) reported effective rates and three (36, 37, 39) reported the occurrence of adverse reactions.

#### 3.6.1 The volume of UFs

Eleven trials showed that acupuncture was superior to the control group in the volume of UFs, but there was high heterogeneity among them (MD −3.89, 95% CI −5.23 to −2.56, *p* < 0.00001, I^2^ = 91%; Supplementary Figure 1). Here, seven trials were divided into two subgroups based on different acupuncture methods and controlled intervention measures. In the subgroup analysis, two trials (studies 2,6) including 124 participants showed that electroacupuncture had better therapeutic effects compared to TCM (MD −6.83, 95% CI −8.79 to −4.86, *p* < 0.00001, I^2^ = 8%; Supplementary Figure 2). Five trials (studies 3,4,5,8,11) including 323 participants showed that traditional acupuncture had better effects compared to TCM (MD −0.66, 95% CI −0.87 to −0.44, *p* < 0.00001, I^2^ = 31%; Supplementary Figure 2). The remaining four trials (studies 1,7,9,10) were not included in subgroup analysis due to significant differences in acupuncture methods or control intervention measures.

#### 3.6.2 Uterine volume

Three trials (studies 7,9,10) involving 208 participants showed that acupuncture has a better therapeutic effect with moderate heterogeneity (MD −16.22, 95% CI −19.89 to −12.55, *p* < 0.00001, I^2^ = 59%; Supplementary Figure 3).

#### 3.6.3 Estrogen levels

In terms of affecting estrogen levels, the therapeutic effect of acupuncture is similar to the control group (MD 3.21, 95% CI – 0.57 to 6.99, *p* = 0.1, I^2^ = 34%; Supplementary Figure 4). These data are sourced from two trials (studies 3,9) involving 124 participants.

#### 3.6.4 The symptom score

A meta-analysis was conducted on four trials (studies 4,8,9,10) with 245 participants, and the results showed high heterogeneity (MD −3.03, 95% CI −3.45 to −2.60, *p* < 0.00001, I^2^ = 96%; Supplementary Figure 5).

#### 3.6.5 Effective rates

Nine trials (studies 1–5,7,8,10,11) compared the efficacy of acupuncture with other therapies by using effective rates. Five trials (studies 3–5,8,11) found that traditional acupuncture was more effective than conventional Chinese medicine, three trials proved that warm acupuncture and moxibustion (studies 1), acupoint catgut embedding (studies 10), and electroacupuncture (studies 2) were more effective than conventional Chinese medicine, and one trial (studies 3) proved that traditional acupuncture was more effective than mifepristone. In all, the results showed that the efficacy of acupuncture was better than that of the control group (RR: 0.19, 95% CI: 0.13 to 0.25, *p* < 0.00001, I^2^ = 0%; Supplementary Figure 6).

#### 3.6.6 Adverse events

Among the three trials that recorded adverse events, one (study 8) did not have any adverse events, while the other (study 10) did not clearly report the specific number of adverse reactions in each group. Only one trial (study 7) reported the specific number and events of adverse reactions, so we did not conduct a meta-analysis of the incidence rate of adverse reactions.

### 3.7 Description of acupuncture regimen

The course of treatment, length of needle retention, and frequency of acupuncture treatment showed diversity in different trials (Supplementary Table 1). Semi-standardized acupuncture protocols were used in two trials (studies 1,5), and standardized methods were used in other trials (studies 2,3,4,6,7,8,9,10,11). The most commonly used acupoints are Guanyuan (RN14), Sanyinjiao (SP6), Zigong (EX-CA1), Xuehai (SP10), Qihai (RN6), Zusanli (ST36), Hegu (LI4), and Taichong (LR3), which have been used more than 5 times. The number of acupoints selected varies from 3 (study 3) to 16 (study 11), with an average of 9.6(SD = 4.2).

Seven trials (studies 3,4,5,7,8,9,11) used traditional acupuncture, two (studies 2,6) used electroacupuncture, one used warm acupuncture and moxibustion (study 1), and one used acupoint catgut embedding (study 10). The frequency of acupuncture is mostly once a day (studies 4,7 8,9), and most patients have been treated for 3 months (studies 1,2,3,6,10,11), with an average duration of 68.7 days (SD = 25.4).

### 3.8 Standard in reporting acupuncture treatment

Supplementary Table 2 lists the summary of acupuncture treatment protocol reports using STRICTA standards. All 11 trials reported the style of acupuncture, provided a reason for treatment and comparator interventions in detail. But the details of needling, such as the number and depth of needles inserted, the responses sought, and the needle type, are not detailed enough to meet the STRICTA standard. All trials reported the acupuncture points used, but in two of them (studies 1,7), the acupuncture points were not fixed. Seven trials (studies 1,2,3,4,6,7,11) reported other interventions besides acupuncture, including moxibustion, waveform setting of electroacupuncture instruments, and medical advice during the treatment period. Three trials (studies 3,4,10) reported on the setting and context of treatment and practitioner background.

### 3.9 Sensitivity analysis

In assessing the uterine size, we performed a sensitivity analysis by removing data from one trial (39) and found a significant reduction in heterogeneity (I^2^ = 0%), suggesting that the source of heterogeneity may be the different acupuncture methods.

In assessing the uterine size, the sensitivity analysis results showed that when the last study (study 10) was removed and only the first three studies (studies 4,8,9) were retained, heterogeneity was low, indicating that the therapeutic effect of acupuncture was better than that of the control group (MD −4.68, 95% CI −5.24 to −4.12, *p* < 0.00001, I^2^ = 0%; Supplementary Figure 7).

## 4 Discussion

### 4.1 Efficacy and safety of acupuncture for UFs

This study reviewed the feasibility, effectiveness, and safety of acupuncture in the treatment of UFs. A total of 11 studies were included in the review and meta-analysis. To our knowledge, this is the first meta-analysis that included studies to solve this specific problem. The preliminary data of the included research shows that acupuncture treatment of UFs has no serious adverse events and is feasible. The sub-group meta-analysis results show that after 1–3 months of treatment, electroacupuncture and traditional acupuncture can reduce the volume of UFs or uterus more effectively than conventional treatment. However, there is not enough high-quality research to conduct subgroup analysis to explore the efficacy of other more diversified acupuncture methods on UFs and evaluate the long-term efficacy.

Study (41) have shown that acupuncture can improve microcirculation and inhibit excessive fibrosis, which may be the effect of treating UFs. However, due to limited clinical trials available, the evidence of acupuncture treatment of UFs is not completely conclusive. Our study comprehensively and systematically reviewed the literature in the databases, identified 11 randomized controlled trials, and provided higher-quality evidence for acupuncture treatment of UFs. The ROB scores of the included trials are all below 9 points. Although according to the high heterogeneity of acupuncture methods and research design, the evidence of using acupuncture to reduce the size of UFs is different, some interesting observations can be found. First of all, each study used the volume of UFs or uterine volume as a therapeutic index, indicating that reducing the volume of UFs is the main treatment goal of patients and doctors in China. Secondly, in approximately half of the studies, patients with TCM syndrome differentiation were recruited, or TCM syndrome differentiation was performed during acupuncture point selection, indicating that acupuncture, as a classic treatment method of TCM, is related to disease types and TCM syndrome types. However, further exploration is needed to determine whether myoma patients can benefit more from syndrome differentiation. Third, UFs are an estrogen-dependent disease. The reduction of estrogen can lead to the reduction of the size of the myoma, this is also the target of western medicine in treating UFs, but acupuncture cannot effectively reduce the estrogen level, which may prove that the mechanism of TCM in treating fibroids is different from western medicine, and the specific molecular pathways affected by acupuncture need further experimental research.

The available research shows that acupuncture is safe in the treatment of UFs. All reported acupuncture-related adverse events were moderate or mild in severity, including dizziness, hot flashes, abdominal pain, etc. All trials indicated normal blood function and normal liver and kidney function, which can help ensure the safety of participants.

In addition, we think it is important to emphasize that acupuncture, as a personalized treatment option, faces some ethical and moral issues as well (42, 43). Here is the premise in the process of diagnosis and treatment, qualified acupuncturists carry out therapeutic operations that are virtually free of side effects and, having informed the patient of the relevant information about the potential risks and benefits prior to the treatment, the patient has the right to decide whether or not to undergo the treatment.

### 4.2 Quality of the evidence

All trials included are effective in terms of methodological quality, but at least one area has been rated as high bias risk. Apart from using sham acupuncture as a controlled study, it is unlikely that blinding procedures will be performed in most trials using an open-label design. Therefore, if the statisticians cannot be blinded, the research results may be affected by selection bias and reduce the credibility of the results.

In addition, the measurement of the results of these trials varies depending on the experiment. As an objective indicator, UFs or uterine volume can directly reflect the curative effect. However, there are still trials that use subjective scales such as symptom scores and psychological status as observation indicators, and most trials do not mention the standardized process of filling out scales, which may affect accuracy to some extent.

### 4.3 Acupuncture treatment regimen

The details of the acupuncture treatment plan vary from study to study. Most included trials fail to report sufficient details to meet all STRICTA criteria, which will hinder readers from evaluating the quality of acupuncture treatment. Syndrome differentiation is commonly used in clinical acupuncture treatment, but this personalized diagnostic method is difficult to replicate and evaluate. Standardized acupuncture treatment plans are still the first choice in acupuncture clinical trials. However, many experiments have proven the effectiveness of dialectical treatment, so it is still necessary to find a balance between standardized treatment and flexible personalized treatment.

In conclusion, future research needs to expand the sample size and conduct a more rigorous and effective design of experiments to evaluate the efficacy and safety of acupuncture in the treatment of UFs. The calculation of sample size should be determined based on standardized formulas and previous research. To improve the quality of methodology, future trials should (1) adopt more standardized syndrome differentiation and acupoint selection methods; (2) use standardized scales as outcome indicators (44, 45); (3) comply with STRICTA standards and CONSORT statements; (4) use appropriate blinding methods; and (5) standardize the quality of researchers and the language used in the study.

### 4.4 Limitations

Due to factors such as acupuncture manipulation and intervention methods in the control group, our review included literature with high heterogeneity. We attempted to reduce heterogeneity through subgroup and sensitivity analysis. First of all, acupuncture techniques (such as traditional acupuncture, electroacupuncture, warm acupuncture and moxibustion, etc.), treatment frequency, and course of treatment are different. These factors may affect the effect measurement and become the source of heterogeneity. Secondly, part of the information in the design of experiments is not comprehensive, such as the specific operation of acupuncture and the background of the researcher are not clear, which greatly limits our evaluation of the quality of the literature. Thirdly, all experiments were conducted in China with strong regional and cultural characteristics, and the risk bias was carefully explained.

## 5 Conclusion

Acupuncture has shown great potential in the treatment of UFs, and it may provide an option for conservative alternative therapy for UFs. It is important to clearly emphasize the practical contribution and significance of this study to the future continuation of clinical practice of acupuncture as a minimally invasive adjuvant therapy for UFs. It provides a robust and feasible reference for relevant clinical trials. In the future, more high-quality randomized controlled trials and long-term follow-ups are needed to better verify acupuncture’s effectiveness and safety in treating UFs.

## Data availability statement

The original contributions presented in the study are included in the article/Supplementary material, further inquiries can be directed to the corresponding author.

## Author contributions

YR: Writing – original draft, Writing – review & editing. JZ: Writing – original draft, Writing – review & editing. WW: Data curation, Writing – review & editing. YY: Data curation, Formal analysis, Writing – review & editing. JW: Visualization, Writing – original draft. YT: Validation, Writing – original draft. YL: Validation, Writing – original draft. XL: Project administration, Supervision, Writing – original draft, Writing – review & editing.

## Footnotes

^1^ https://training.cochrane.org/handbook/archive/v5.0.0/
