# Supplementary material for: Should acupuncture become a complementary therapy in the treatment of uterine fibroid: a systematic review and meta-analysis of randomized controlled trials
Source: Front Med (Lausanne). 2023 Dec 13;10:1268220. doi: 10.3389/fmed.2023.1268220 (PMC10751827; doi:10.3389/fmed.2023.1268220)
Supplement: Supplementary file 1 [file Data_Sheet_1.docx]

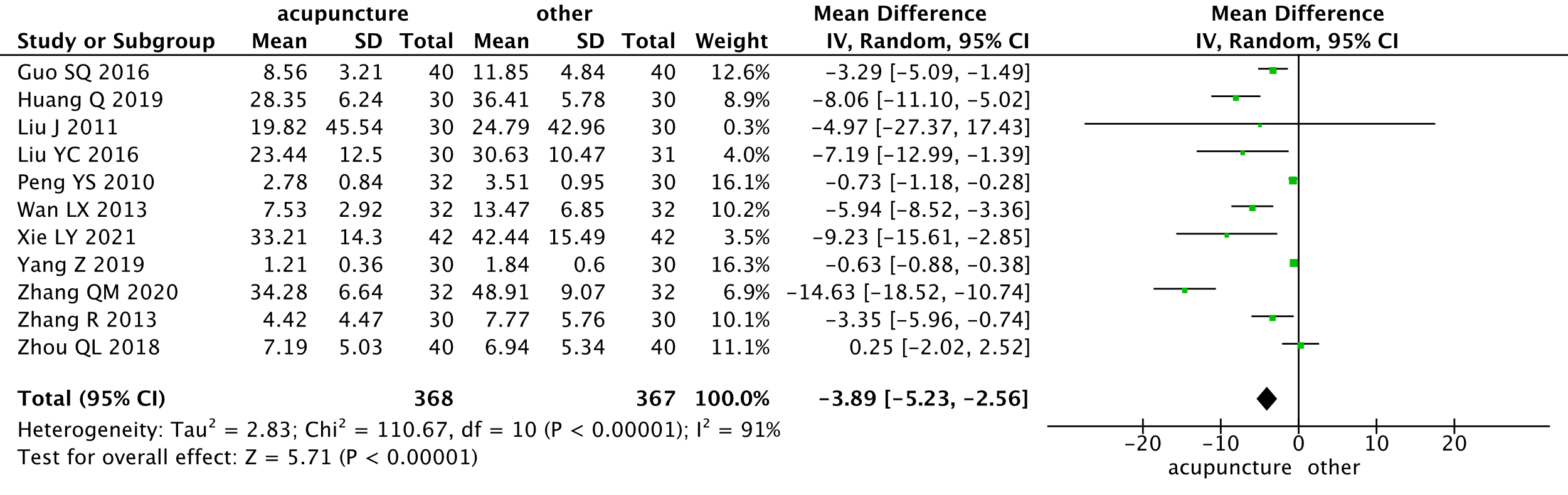


**Supplemental Figure. 1** Forest plot of included trials on the volume of UFs.


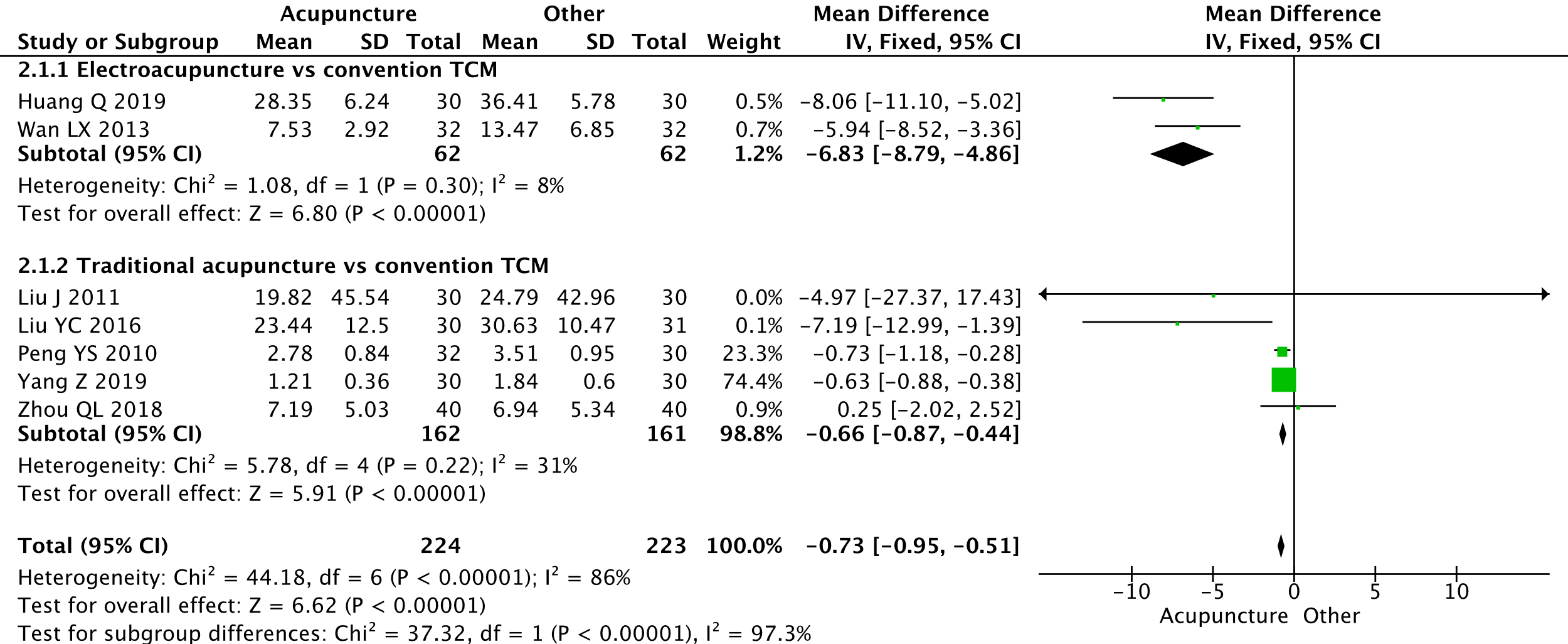


**Supplemental Figure. 2** Forest plot of subgroup analysis on the volume of UFs.


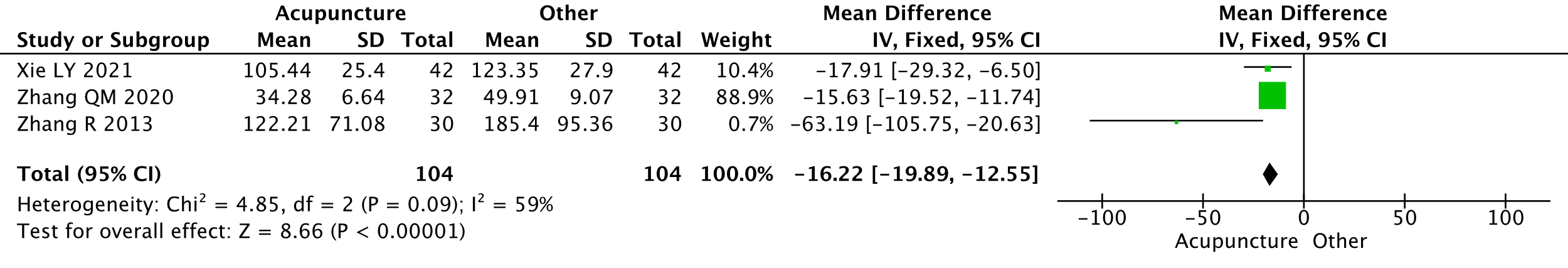


**Supplemental Figure. 3** Forest plot of included trials on the volume of uterine.


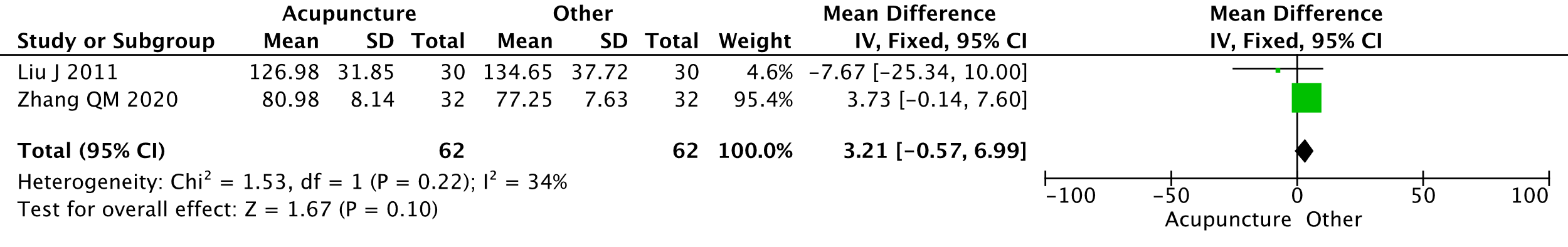


**Supplemental Figure. 4** Forest plot of included trials on estrogen level.


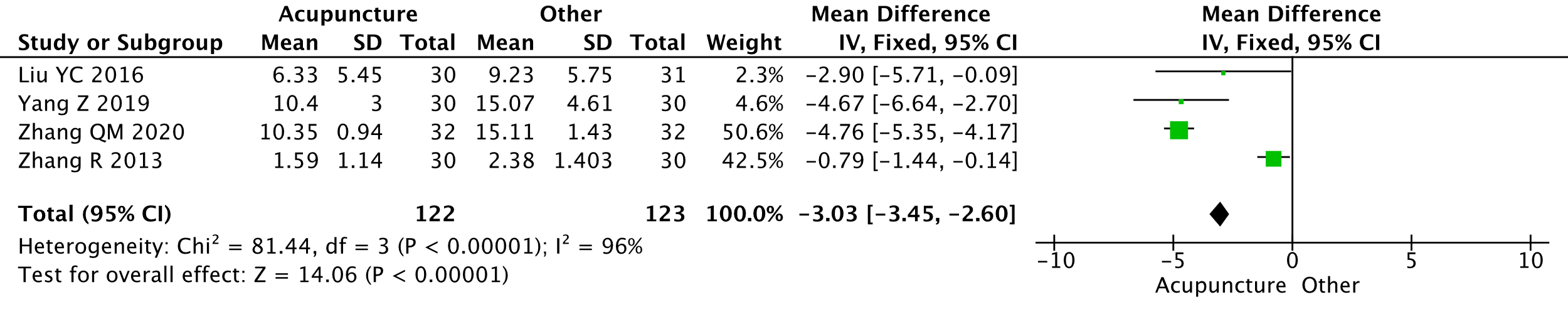


**Supplemental Figure. 5** Forest plot of included trials on symptom score.


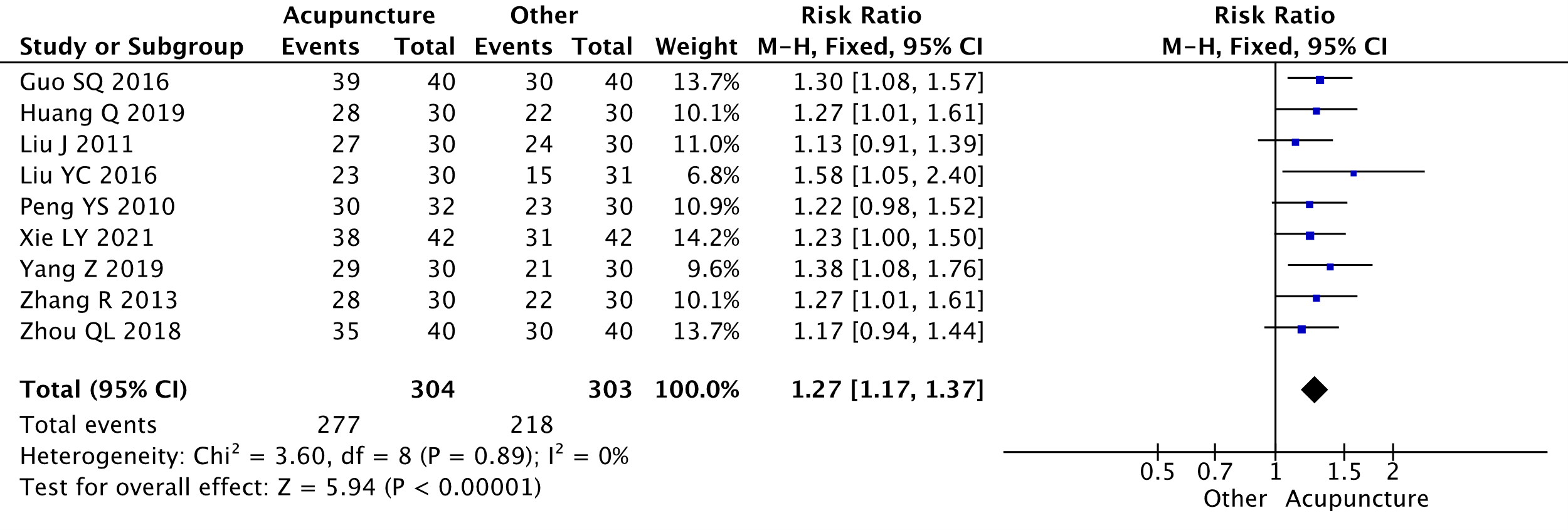


**Supplemental Figure. 6** Forest plot of included trails on effective rate.


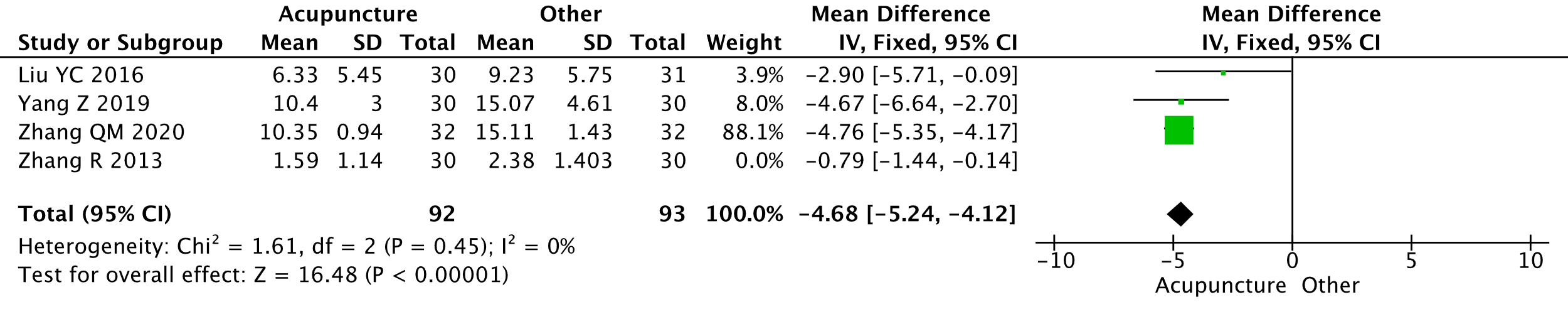


**Supplemental Figure. 7** Forest plot of sensitivity analysis on symptom score.

**Supplemental Table 1**

| Summary of acupuncture treatment protocol using the STRICTA criteria. | | | | |  |
| --- | --- | --- | --- | --- | --- |
| **No.** | **1st Author (Year)** | **Acupuncture type** | **Acupuncture protocol** | **Acupoints** | **Treatment regimen** |
| 1 | Guo 2016 | WAM | Semi- standardized | Acupuncture 12: 5 fixed points, guanyuan(RN4), sanyinjiao(SP6), xuehai(SP10),zigong(EX-CA1);7 additional points, hegu(LI4), taichong(LR3), fenglong(ST40), yinlingquan(SP9), ciliao(BL32), qihai(RN6), taixi(KI3). | 1 month/course of treatment; 3 courses totally |
| 2 | Huang 2019 | EA | standardized | 9: qihai(RN6), zigong(EX-CA1), guanyuan(RN4), zusanli(ST36), xuehai(SP10), zhongji(RN3), sanyinjiao(SP6), neiguan(PC6), hegu(LI4). | three times weekly for 3 monthes;30 min/session |
| 3 | Liu 2011 | TA | standardized | 3: hegu(LI4), taichong(LR3), sanyinjiao(SP6). | once a day for one week and once a week for the residual; 3 monthes totally |
| 4 | Liu 2016 | TA | standardized | 11: guanyuan(RN4), zigong(EX-CA1), zhongji(RN3), sanyinjiao(SP6), tianshu(ST25), xuehai(SP10), qihai(RN6), hegu(LI4), guilai(ST29), taichong(LR3), yinlingquan(SP9). | once a day;2 month/course of treatment; 3 courses totally;30 min/session |
| 5 | Peng 2010 | TA | Semi- standardized | Acupuncture 11: 3 fixed points,tianshu(ST25), guanyuan(RN4), zigong(EX-CA1);8 additional points, neiguan(PC6), shenmen(HT7), zhigou(SJ6), shangjuxu(ST37), yanglingquan(SP9), taichong(LR3); xuehai(SP10); sanyinjiao(SP6). | 2 monthes totally |
| 6 | Wan 2013 | EA | standardized | 8: zigong(EX-CA1), guanyuan(RN4), xuehai(SP10), sanyinjiao(SP6), yinlingquan(SP9), diji(ST4), hegu(LI4), ashi point(unfixed). | 1/day for 15days and 7days rest, periodicity; 3 month totally; 20 min/session |
| 7 | Xie 2021 | TA | standardized | 7: 3 fixed points, tianshu(ST25), guanyuan(RN4), zigong(EX-CA1);4 additional points, qihai(RN6), zusanli(ST28), xuehai(SP10), zhigou(SJ6). | daily for 30 days; 30-45 min/session |
| 8 | Yang 2019 | TA | standardized | Acupuncture 14: 4 fixed points, henggu(KI11), qugu(RN2), zigong(EX-CA1), sanyinjiao(SP6);10 additional points, zhongji(RN3), guanyuan(RN4), qihai(RN6), gongsun(SP4), xuehai(SP10), diji(ST4), fenglong(ST40), zusanli(ST36), shuidao(ST28), guilai(ST29). | daily for 42 days; 30min/session |
| 9 | Zhang 2020 | TA | standardized | Acupuncture 12: 3 fixed points, sanyinjiao(SP6), henggu(KI11), zigong(EX-CA1);9 additional points, zusanli(ST36), guanyuan(RN4), qihai(RN6), gongsun(SP4), zhongji(RN3), diji(SP8), shuidao(ST28), fenglong(ST40), guilai(ST29). | daily for 42 days; 30min/session |
| 10 | Zhang 2013 | CET | standardized | 3: sanyinjiao(SP6), guanyuan(RN4), zigong(EX-CA1). | once a month; 3 times totally |
| 11 | Zhou 2018 | TA | standardized | Acupuncture 16: 8 fixed points, Ganshu(BL18), shenshu(BL23), ciliao(BL32), guanyuan(RN4), qihai(RN6), tianshu(ST25), shuidao(ST28), zigong(EX-CA1);8 additional points, zusanli(ST36), taichong(LR3), sanyinjiao(SP6), taixi(KI3), xuehai(SP10), taichong(LR3), yinlingquan(SP9), taibai(SP3). | three times a week; 40 sessions; 20min/session |
| Abbreviations: *WAM* warm acupuncture and moxibustion; *EA* Electroacupuncture; *TA* traditional acupuncture; *CET* Catgut-embedding therapy | | | | | |

**Supplemental Table 2**

| Evaluation of acupuncture treatment regimen in the included studies using STRICTA criteria. | | | | | | | | | | | | | | |
| --- | --- | --- | --- | --- | --- | --- | --- | --- | --- | --- | --- | --- | --- | --- |
| **No.** | **1st Author (Year)** | **Acupuncture rationale** | **Details of needling** | | | | | | | **Treatment regimen** | **Other treatment components** |  |  |  |
|  |  |  | **No. of needle inserted** | **Points used** | **Depth of insertion** | **Response sought** | **Needle stimulation** | **Needle retention** | **Needle type** |  | **Other interventions** | **Setting and context of treatment** | **Practitioner background** | **Control or comparator interventions** |
| 1 | Guo 2016 | Y | NR | Ya | NR | NR | Y | NR | NR | Yb | Y | NR | NR | Y |
| 2 | Huang 2019 | Y | Y | Y | NR | Y | Y | Y | Y | Y | Y | NR | NR | Y |
| 3 | Liu 2011 | Y | Y | Yb | NR | NR | Y | NR | NR | Y | Y | Y | Y | Y |
| 4 | Liu 2016 | Y | Y | Y | NR | Y | Y | Y | Y | Y | Y | Y | Y | Y |
| 5 | Peng 2010 | Y | NR | Yb | NR | NR | Y | NR | NR | Yb | NR | NR | NR | Y |
| 6 | Wan 2013 | Y | Y | Y | NR | Y | Y | Y | Y | Y | Y | NR | NR | Y |
| 7 | Xie 2021 | Y | Y | Ya | Y | NR | Y | Y | NR | Y | Y | NR | NR | Y |
| 8 | Yang 2019 | Y | NR | Yb | NR | Y | Y | Y | NR | Y | NR | NR | NR | Y |
| 9 | Zhang 2020 | Y | NR | Yb | NR | Y | Y | Y | NR | Y | NR | NR | NR | Y |
| 10 | Zhang 2013 | Y | Y | Y | Y | NA | Y | NA | Y | Y | NR | Y | Y | Y |
| 11 | Zhou 2018 | Y | Y | Y | NR | Y | Y | Y | Y | Y | Y | NR | NR | Y |
| Note: NA, not applicable; NR not reported; Y, reported; Ya, reported but points were not fixed; Yb, reported but the total numbers were unclear. | | | | | | | | | | | | | | |
